# Multiparametric MRI radiomics nomogram predicts synchronous distant metastasis in rectal cancer

**DOI:** 10.1038/s41598-026-35973-w

**Published:** 2026-01-19

**Authors:** Hao Jiang, Wei Guo, Xue Lin, Zhuo Yu, Yudie Qin, Zhongqi Sun, Hongbo Hu, Jinping Li, Linhan Zhang, Qiong Wu, Huijie Jiang

**Affiliations:** 1 Department of Radiology, The Second Affiliated Hospital of Harbin Medical University, No. 246, Xuefu Road, Nangang District, Harbin, 150086 China; 2 Department of PET/CT-MR, Harbin Medical University Cancer Hospital, Harbin, China; 3 Hangzhou Lin ping research medical film technical service studio, Hangzhou, China; 4 Department of Nuclear Medicine, The First Affiliated Hospital of Harbin Medical University, Harbin, China

**Keywords:** Magnetic resonance imaging, Rectal cancer, Synchronous distant metastasis, Nomogram, Cancer imaging, Rectal cancer, Metastasis

## Abstract

This study aimed to evaluate the utility of a multiparametric MRI-based radiomics nomogram for identifying patients with rectal cancer (RC) at high risk of synchronous distant metastasis (SDM). A fusion feature selection strategy, which combined univariate analysis with three machine learning algorithms, was employed to optimize predictive signatures from the 1,688 radiomics features extracted using PyRadiomics. A retrospective cohort of 169 RC patients (stratified into training and test sets at an 8:2 ratio, *n* = 134/35) was analyzed. Among these, 48.5% (82/169) presented with SDM. Following the screening process, four clinical characteristics were selected. Feature selection yielded eight features from diffusion-weighted (DW) images, eight from T2-weighted (T2W) images, and six from the combined radiomics model (integrating DW and T2W phases). The clinical-radiomics nomogram demonstrated superior predictive performance over standalone clinical or radiomics models, achieving areas under the curve (AUC) of 0.93 (95% CI: 0.89–0.96) and 0.94 (95% CI: 0.79–0.97) in the training and test cohorts, respectively, with balanced sensitivity (0.85–0.88) and specificity (0.86–0.89). The calibration plots demonstrated alignment between the nomogram’s predictions and actual outcomes. Decision curve analysis (DCA) indicated that the nomogram model provided the highest net benefit across both the training and test sets, outperforming the standalone clinical and radiomics models. Consequently, this MRI-based radiomics nomogram could assist in the preoperative identification of RC patients at high SDM risk, thereby optimizing clinical management strategies.

**Supplementary Information:**

The online version contains supplementary material available at 10.1038/s41598-026-35973-w.

## Introduction

Rectal cancer (RC) ranks as the third leading cause of cancer-related morbidity and mortality worldwide^1^. Despite advancements in total mesorectal excision and neoadjuvant chemoradiotherapy, which have significantly reduced local recurrence rates^2,3^, distant metastasis remains the primary cause of treatment failure for RC patients^4,5^. Surgical excision serves as the mainstay of treatment for early metastatic disease. Surgical resection of colorectal liver or lung metastasis can improve 5-year survival rates to 56.2% and 52.2%, respectively^6,7^. Thus, given this potential benefit, the preoperative identification of RC patients at high risk for synchronous distant metastasis (SDM) is crucial for personalized management. However, the first-line imaging method for RC liver metastasis may lead to either missed metastases or unnecessary operations^8^. MRI is increasingly becoming the standard modality for detecting liver metastases, and contrast-enhanced liver MRI is recommended for all patients scheduled for local therapy of colorectal liver metastases. These approaches aim to improve early detection, enhance therapeutic efficacy, and potentially increase survival rates. Nevertheless, few studies have focused on predicting SDM in RC specifically, and no consensus exists on the matter.

MRI-based radiomics is a non-invasive, high-throughput post-processing technique that extracts a vast number of quantitative features from standard medical images^9–11^. Furthermore, numerous studies have successfully applied conventional radiomics to MRI for predicting chemotherapy efficacy^12–14^, metastasis^5,15^, prognosis^16,17^, and molecular typing^18–20^ in patients with rectal cancer. Our previous experience confirmed that MRI staging-based radiomics models provide a relevant predictive tool for identifying the oncological behavior of pCR after nCRT. Moreover, while recent research by Zhao et al. indicates that the tumor stroma ratio (TSR) from colonoscopy biopsies can independently stratify distant metastasis risk in patients with LARC, MRI radiomic analysis could serve as an ideal alternative for patients in whom a colonoscopy biopsy is unavailable^21^. Previous studies have demonstrated the feasibility of predictive nomograms for assessing the probability of synchronous liver, lung, and bone-distant disease in 46,785 RC patients. Although these results were promising, the proposed nomograms relied on clinical and pathologic features, the latter of which are only available postoperatively. This limitation restricts their utility in guiding preoperative management strategies^22^. Consequently, there is a clear need for preoperative, non-invasive predictive models to inform early decision-making and optimize personalized management for RC patients.

Machine learning (ML) has emerged as a powerful tool for medical image analysis and diagnosis classification, enabling the decoding of complex patterns from high-dimensional data such as radiomics features. Support Vector Machines (SVM) are renowned for their robust margin-maximization principle and flexibility via kernel functions, making them particularly effective among ML algorithms for handling small-to-medium-sized datasets with high-dimensional features. In this context, SVM is particularly suited for radiomics-based classification tasks due to its resilience to overfitting and ability to handle non-linear relationships via kernels. Although alternative classifiers may offer competitive performance, SVM was prioritized for its interpretability, computational efficiency, and proven success in similar radiomics studies predicting oncological outcomes^23–26^. This decision was guided by the need to balance model complexity with generalizability, a key consideration given the moderate sample size of this study.

To the best of our knowledge, few studies have investigated the feasibility of a multiparametric MRI-based radiomics nomogram for predicting SDM in rectal cancer patients. While Liu et al. demonstrated that T2-weighted images (T2WI)-based radiomics analysis helps predict SDM of RC^27^, our work extends this by integrating multiparametric data into a nomogram. However, diffusion-weighted imaging (DWI), another proper and routinely used sequence that reflects tumor cellularity, was not incorporated into their radiomics analysis. Given previous research findings^28,29^, we hypothesize that including DWI could enhance the predictive performance of radiomics analysis by providing complementary information to improve the accuracy of SDM prediction. This study aimed to develop and validate a predictive nomogram based on T2WI and DWI MRI radiomics to preoperatively identify RC patients at high risk of synchronous distant metastasis (SDM), thereby aiding clinical decision-making and guiding personalized management.

## Methods

### Patient

**Fig. 1:**
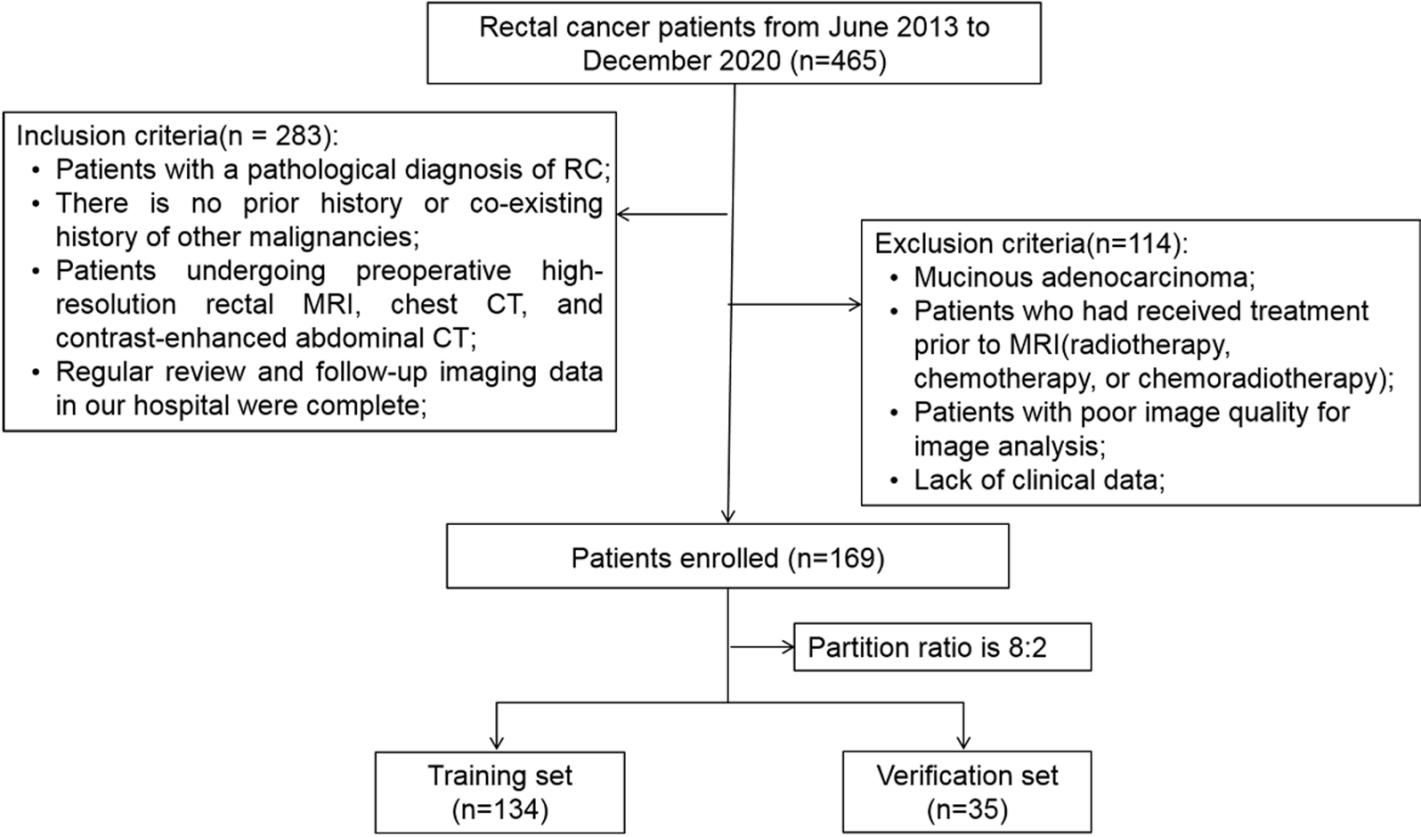
Flow chart of patients’ recruitment pathway.

**Fig. 2:**
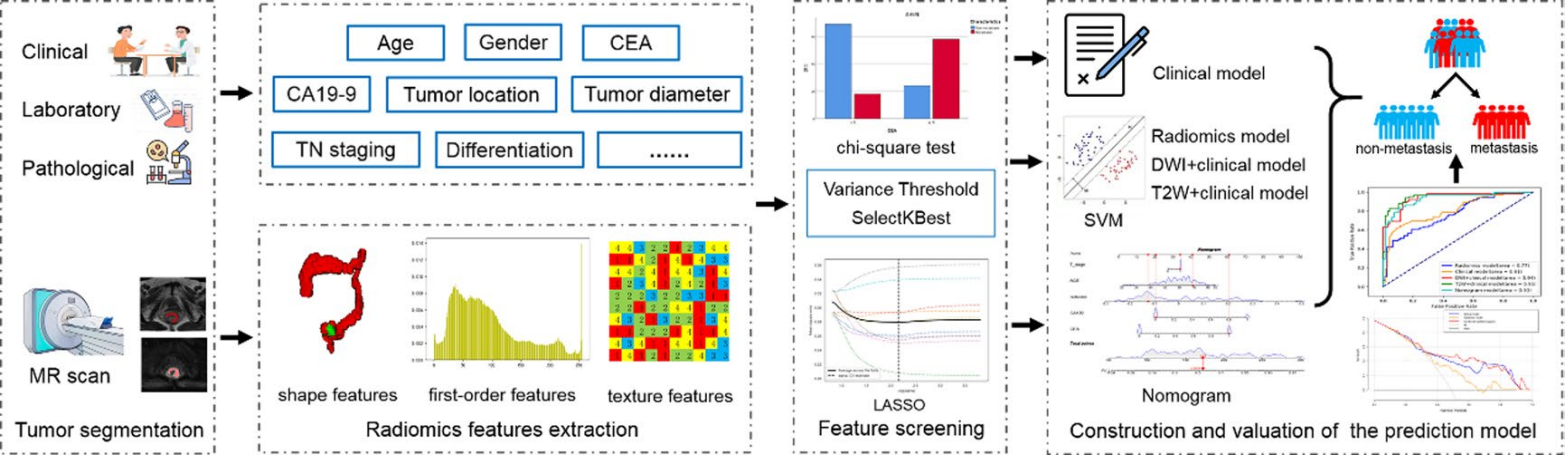
The workflow of the study.

This retrospective study was approved by the Institutional Review Board of The Second Affiliated Hospital of Harbin Medical University (Approval No. KY2025-068), and the requirement for informed consent was waived. All patient data were anonymized before analysis. We retrospectively reviewed the clinicopathological and imaging data of 465 rectal cancer (RC) patients diagnosed by endoscopic biopsy or surgical pathology between January 2013 and December 2020. From this cohort, 283 patients who had undergone high-resolution pretreatment rectal MRI, unenhanced chest CT, and contrast-enhanced abdominal CT examinations were ultimately enrolled. The collected clinical data included age, gender, pre-treated carcinoembryonic antigen (CEA) and carbohydrate antigen 19 − 9 (CA19-9) levels, tumor diameter, tumor location, as well as T and N stages predicted by MRI. Based on the exclusion criteria detailed in Figs. 1 and 114 patients were excluded. Following exclusions, the remaining 169 patients were included and randomly divided into a training dataset (*n* = 134) and a test dataset (*n* = 35) at an 8:2 ratio. The overall workflow of the study is presented in Fig. 2.

### Imaging acquisition

Rectal MRI was performed for all patients using a GE Discovery MR750w 3.0T MRI scanner equipped with a 32-channel phased array coil, with patients in the supine position. For preparation, patients were instructed to follow a low-residue diet one day prior and to fast for 4–6 h before the examination. The imaging sequences consisted of the following: oblique axial, coronal, and sagittal T2-weighted images (T2WI) and axial diffusion-weighted imaging (DWI, b values of 0 and 800 s/mm^²^). The detailed protocols are summarized in Appendix 1. All patients underwent CT imaging of the chest and abdomen using a dual-source 64-MDCT scanner (Somatom Definition Flash, Siemens Healthineers).

### Qualitative image evaluation

Two radiologists (H.H. and J.L., with over 10 years of experience in abdominal radiology diagnosis) examined all MRI data images. They evaluated the T and N stages as defined by the American Cancer Federation’s TN Staging System for Rectal Cancer, Version 8. Two other radiologists (H.J. and L.Z., with over 5 years’ experience in chest and abdominal radiology diagnosis) consistently analyzed chest and abdominal CT images for the assessment of distant metastasis. All radiologists knew that these tumors were biopsy-confirmed adenocarcinomas of the rectum, but they did not know the histopathological stage of the patient. When consensus could not be reached, a final opinion was sought from an additional experienced radiologist (H.J.J., with over 20 years of experience in chest and abdominal diagnosis).

### Quantitative image evaluation

#### Image segmentation

After the two radiologists responsible for qualitative MR evaluation identified the tumor region on oblique axial T2WI and DWI, they independently and manually segmented the entire region of interest (ROI) of the primary tumor using a free, open-source software package (ITK-SNAP, version 3.4.0, www.itksnap.org). The delineations encompassed the surrounding structures and bundles suspected of tumor infiltration while excluding the non-invaded rectal wall and the intestinal lumen. To ensure the reproducibility and stability of the segmentation outcomes, 30 samples were randomly selected one month later, and two doctors conducted independent segmentations again. Subsequently, the intraclass correlation coefficient (ICC) was used to assess intra-observer and inter-observer reproducibility, with a value of > 0.75 considered robust.

#### Image feature extraction and selection

All procedures were performed according to the Image Biomarker Standardization Initiative (IBSI) standards^30^. The PyRadiomics package (v3.0.1; http://www.radiomics.io/pyradiomics.html, IBSI-compliant) extracted 1,688 radiomic features, including first-order features, shape features, texture features, and wavelet features from 10 image types. These image types are the original image, Laplacian of Gaussian (LoG, based on the Simple ITK function), Wavelet (using the PyWavelets package), Square, Square Root, Logarithm, Exponential, Gradient, Local Binary Pattern (2D), and Local Binary Pattern (3D).

For feature screening, we sequentially applied Variance Threshold (to remove low-variance features, < 0.8), SelectKBest, and Least Absolute Shrinkage and Selection Operator (LASSO). Low-variance features were eliminated because their minimal value fluctuation contributes little to outcome discrimination. For the variance threshold method, values with variances less than the variance threshold (0.8) are removed. The SelectKBest method is based on the chi-square test and retains the features with *P* < 0.05. The chi-square test is a method for measuring the degree of deviation between the actual observed value of the statistical sample and the theoretically inferred value. The LASSO regression uses the least squares loss function (i.e., L2 loss) in combination with an L1 regularization term. Finally, a radiomics model was built based on Support Vector Machines (SVM).

#### Development and valuation of the prediction model

To construct the clinical model, univariate analysis was performed to identify potential risk factors. Those with statistical significance (*P* < 0.05) were subsequently incorporated into the model. According to the above process, features were extracted from different phases of images, and the pooling features of the two phases were screened. Then, based on these screened features, the DWI model, the T2W model, and the radiomics models were constructed. The three clinical-radiomics models (DWI + clinical model, T2W + clinical model, and nomogram) were constructed using the selected pooled features from both phases and independent clinical risk factors.

To evaluate the performance of these models, five indicators were selected for evaluation: receiver operating characteristic (ROC), area under the curve (AUC), accuracy, sensitivity, specificity, and 95% confidence interval (CI).

### Statistical analysis

Continuous variables were expressed as means ± standard deviations (SD) or medians with interquartile ranges (IQR), and analyzed using an independent-sample t - test or Mann–Whitney U test. Categorical variables were presented as counts or percentages and were analyzed using Pearson’s Chi-square test or Fisher’s exact test as appropriate. ROC curves were plotted, and the AUC was computed to evaluate the diagnostic performance of each model. Decision curve analysis (DCA) was conducted to compare the differences in net benefits among the different models. SPSS 26.0 statistical software was utilized to analyze the data. A test standard of *P* = 0.05 was adopted.

All experiments are completed using the PyCharm program. The programming language used is Python (v. 3.6), mainly utilizing packages such as sklearn, numpy, scipy, and pandas.

## Results

### Patient characteristics

A total of 169 patients were included in the analysis. Baseline clinical characteristics are presented in Table 1. No significant difference was detected in the data distribution between the training set and the test set (*P* = 0.90). Additionally, there were no significant differences between the two groups in terms of gender, tumor location, tumor diameter, and degree of differentiation (*P* > 0.05). CEA and CA19-9 levels were significantly higher in the metastasis group than in the non-metastasis group (*P* < 0.001). Regarding the MR staging of RC, significant differences were observed for T staging between the two groups (*P* = 0.040).

**Table 1:** Characteristics of patients and associations with synchronous distant metastasis.

Characteristics	Non-metastasis (*n* = 87)	Metastasis (*n* = 82)	χ^2^/t	*P* value
Age, mean ± SD, years	57.9 ± 11.8	62.3 ± 8.4	−2.82	0.005**
Gender (%)				
Male	62 (71.3)	53 (64.6)		
Female	25 (28.7)	29 (35.4)	0.85	0.356
CEA, µg/L				
< 5	69 (79.3)	24 (29.3)		
≥ 5	18 (20.7)	58 (70.7)	42.72	< 0.001***
CA19-9, U/ml				
< 37	78 (89.7)	47 (57.3)		
≥ 37	9 (10.3)	35 (42.7)	22.92	< 0.001***
Tumor diameter, mean ± SD, cm	4.3 ± 1.6	4.6 ± 1.2	−1.73	0.086
Tumor location (%)				
Proximal rectum	31 (35.6)	23 (28.1)		
Middle rectum	50 (57.5)	48 (58.5)		
Distal rectum	6 (6.9)	11 (13.4)	2.55	0.279
Degree of differentiation (%)				
Well	7 (8.0)	10 (12.2)		
Moderate	74 (85.1)	64 (78.0)		
Poor	6 (6.9)	8 (9.8)	1.39	0.498
T staging (%)				
T1-2	14 (16.1)	5 (6.1)		
T3-4	73 (83.9)	77 (93.9)	4.23	0.040*
N staging (%)				
N0	23 (26.4)	13 (15.9)		
N1-2	64 (73.6)	69 (84.1)	2.85	0.093

*, *P* < 0.05; **, *P* < 0.01; ***, *P* < 0.001. CEA, carcinoembryonic antigen; CA19-9, carbohydrate antigen 19 − 9.

Among the patients, 82 (48.5%) were confirmed to have SDM. The metastatic locations were the liver (*n* = 32), lung (*n* = 19), bone (*n* = 8), peritoneum (*n* = 4), both the liver and lung (*n* = 15), both the liver and distant lymph nodes (*n* = 2), and simultaneous involvement of the liver, lung, and peritoneum (*n* = 2). Of these cases, 18 were confirmed by histopathology (via surgery or biopsy); 29 were diagnosed based on the typical appearance of multifocal metastatic disease or multimodal radiological features; and the remaining 35 were confirmed during follow-up, defined by either an increase in the number or size of lesions without treatment, or stability or reduction in the number or size of lesions following appropriate treatment.

### Features selection, construction, and valuation of the radiomics model

After feature screening, DW phase images retained eight features; T2W phase images retained eight features; the Radiomics model (DW phase and T2W phase images) retained six features. Among these, there are four features from DW phase images and two features from T2W phase images. The selected radiomics features and their corresponding weighted coefficients are shown, respectively, in Supplementary Table S1. The Rad-score calculation formulas of the three models are presented in the Supplementary Materials.

**Fig. 3:**
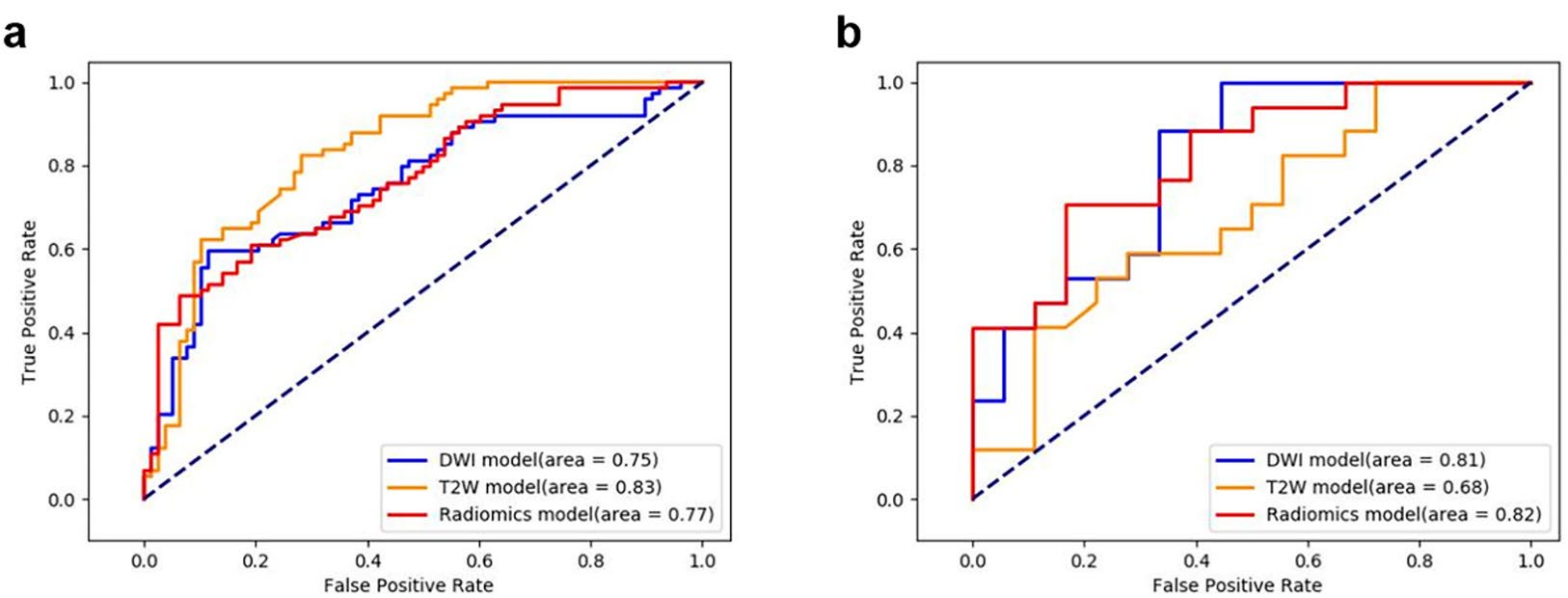
Performance of models based on different phase images. (**a**) Training set, (**b**) Test set.

Regarding the retained characteristics of each phase, the SVM model was chosen to build the radiomics model for each phase. The key SVM hyperparameters employed were as follows: a sigmoid kernel, C = 200, with probability set to True, and class_weight set to ‘balanced’. The experimental results demonstrate that the three models exhibit good discriminative ability for SDM (Table 2; Fig. 3). Specifically, on the training set, the T2W model performed the best. Its AUC, accuracy, sensitivity, specificity, and 95% CI were 0.83, 0.76 (102/134), 0.81 (53/65), 0.71 (49/69), and 0.72–0.89, respectively. On the test set, the Radiomics model performed the best. Its AUC, accuracy, sensitivity, specificity, and 95% CI are 0.82, 0.71 (25/35), 0.76 (13/17), 0.67 (12/18), and 0.62–0.86, respectively.

**Table 2:** Performance of models based on different phase images.

Classifiers	Training set					Test set				
	AUC	ACC	SEN	SPE	95%CI	AUC	ACC	SEN	SPE	95%CI
DWI model	0.75	0.65	0.71	0.69	0.65–0.84	0.81	0.71	0.76	0.67	0.59–0.83
T2W model	0.83	0.76	0.81	0.71	0.72–0.89	0.68	0.60	0.59	0.61	0.44–0.73
Radiomics model	0.77	0.66	0.66	0.67	0.70–0.83	0.82	0.71	0.76	0.67	0.62–0.86

AUC, area under the curve; ACC, accuracy; SEN, sensitivity; SPE, specificity; CI, confidence interval.

### Construction of the clinical-radiomics signature

**Fig. 4:**
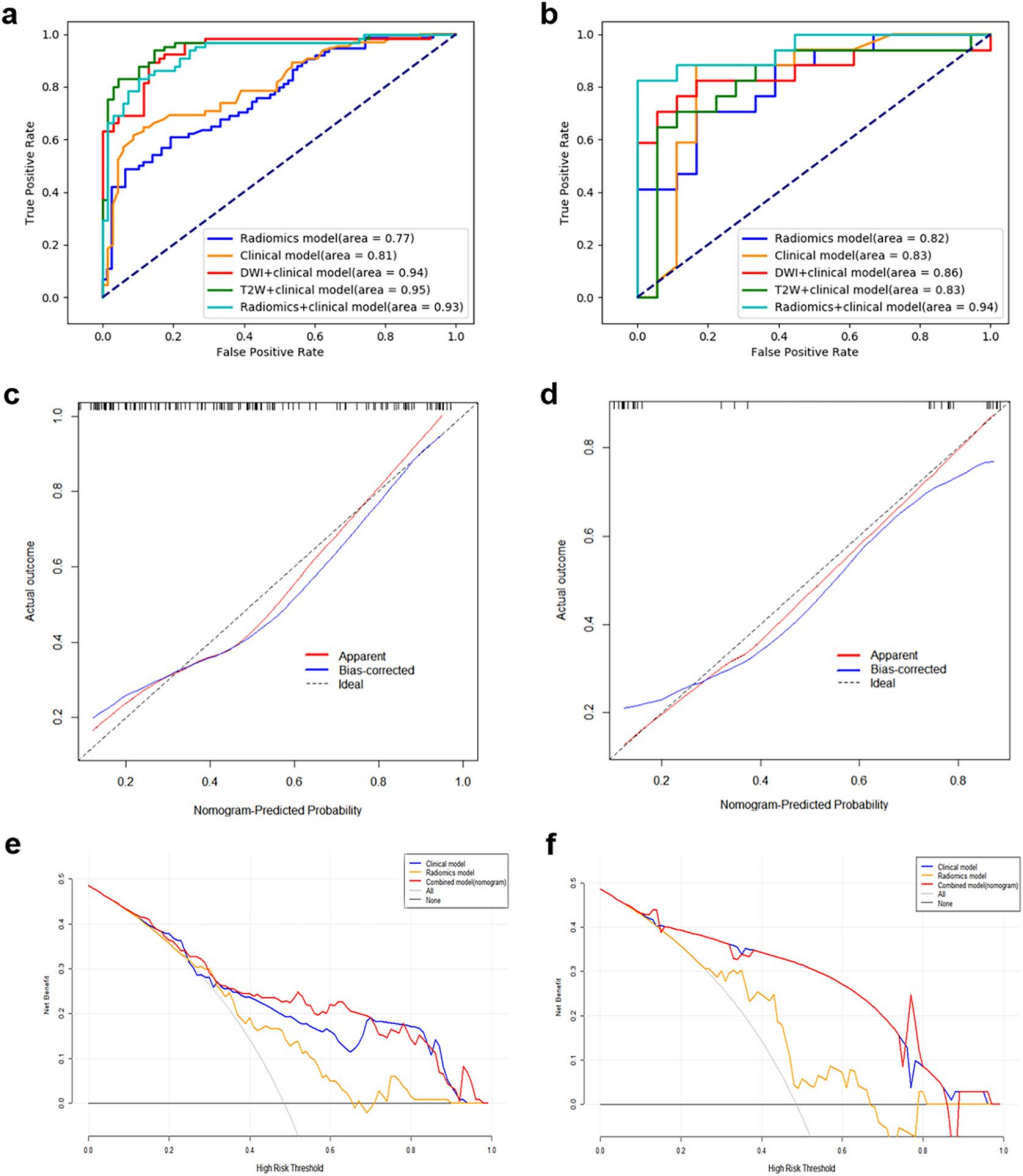
Comprehensive evaluation of prediction models in the training and test sets. Receiver operating characteristic (ROC) curves of the radiomics model, clinical model, DWI + clinical model, T2W + clinical model and nomogram model in the training set (**a**) and test set (**b**). Calibration curves of the nomogram model in the training set (**c**) and test set (**d**). Decision curve analysis (DCA) comparing the net benefit of the clinical, radiomics, and nomogram models in the training set (**e**) and test set (**f**).

**Fig. 5:**
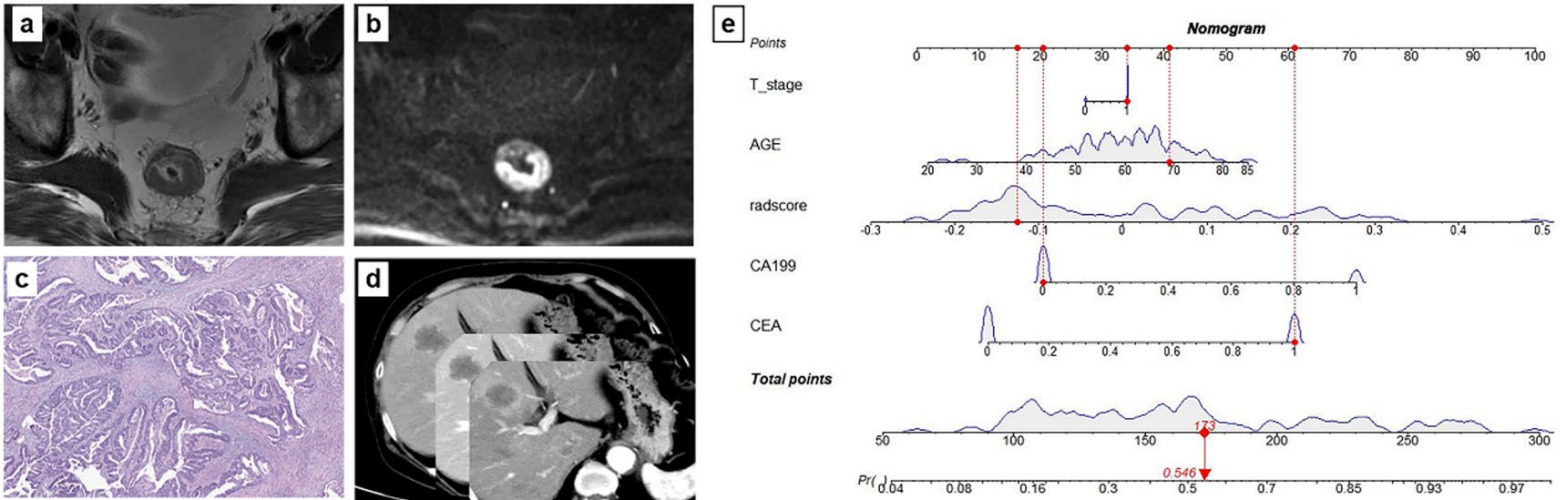
The developed clinical-radiomics nomogram. A 69 year old male underwent preoperative MR imaging, (**a**) T2W imaging demonstrates a moderately hyperintense mass in the rectum, penetrating the rectal muscle layer (T3 stage), (**b**) DW imaging reveals a mass with a heterogenous high signal (restricted diffusion), CEA: 13.2 µg/L, CA19-9: 26.8 U/ml, the radscore: −0.122, (**c**) HE staining (100×) revealing that the tumor had invaded the surrounding adipose tissue of the rectum, (**d**) Liver synchronous distant metastasis occurred 2.5 months after surgery, (**e**) Vertical lines of each variable were drawn and total points: (34 + 41 + 16 + 21 + 61 = 173).

The clinical model developed using four independent risk factors (CEA, age, CA19-9, and T stage) demonstrated AUCs of 0.81 and 0.83 in the training and test datasets, respectively (Fig. 4a and b). Combining clinical factors and radiomics characteristics of different sequence images, we established three clinical-radiomics models, namely the DWI + clinical model, the T2W + clinical model, and the nomogram (radiomics + clinical) model (Fig. 5). In terms of indicators, the nomogram model has the best discriminative performance on SDM (Table 3; Fig. 4a and b). On the training set, the AUC, accuracy, sensitivity, specificity, and 95% CI of the nomogram model are 0.93, 0.85 (114/134), 0.85 (55/65), 0.86 (59/69), and 0.89–0.96 respectively. On the test set, the AUC, accuracy, sensitivity, specificity, and 95% CI of the nomogram model are 0.94, 0.89 (31/35), 0.88 (15/17), 0.89 (16/18), and 0.79–0.97, respectively. To strengthen the validation, we performed supplementary 5-fold cross-validation (results presented in Supplementary Table S2), which demonstrated stable model performance.

**Table 3:** Performance of radiomics model, clinical model, DWI + clinical model, T2W + clinical model, and nomogram.

Classifiers	Training set					Test set				
	AUC	ACC	SEN	SPE	95%CI	AUC	ACC	SEN	SPE	95%CI
Radiomics model	0.77	0.66	0.66	0.67	0.70–0.83	0.82	0.71	0.76	0.67	0.62–0.86
Clinical model	0.81	0.70	0.69	0.71	0.74–0.86	0.83	0.83	0.82	0.83	0.72–0.92
DWI + clinical model	0.94	0.87	0.88	0.87	0.90–0.97	0.86	0.80	0.71	0.89	0.68–0.89
T2W + clinical model	0.95	0.88	0.88	0.88	0.91–0.98	0.83	0.74	0.82	0.67	0.62–0.86
Nomogram model	0.93	0.85	0.85	0.86	0.89–0.96	0.94	0.89	0.88	0.89	0.79–0.97

AUC, area under the curve; ACC, accuracy; SEN, sensitivity; SPE, specificity; CI, confidence interval.

### Clinical utility and interpretability

The performance of the predictive model was further evaluated from a clinical utility perspective. The calibration plots (Fig. 4c and d) were consistent between the probabilities predicted by the clinical radiomics model and the observed probabilities. The confusion matrices provided a detailed breakdown of the model’s classification performance (Supplementary Fig. S1). Decision curve analysis (Fig. 4e and f) showed that the nomogram model achieved the highest net benefit compared with the clinical model and the radiomics model on the training and test sets.

**Fig. 6:**
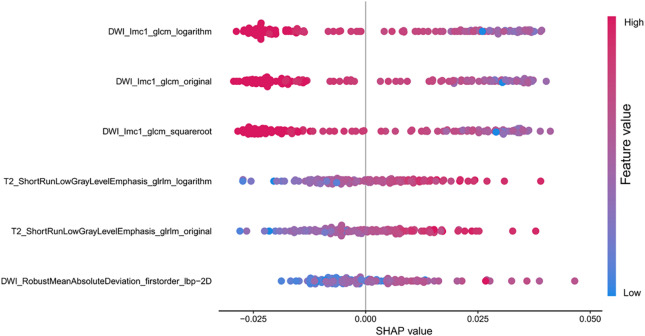
Feature importance and impact direction from SHAP analysis. The top six features determining the model’s prediction are shown. Red (high value) and blue (low value) points indicate how each feature value pushes the prediction towards a higher or lower risk of metastasis, respectively.

The SHAP summary plot (Fig. 6) revealed the top six features that most significantly influenced the model’s predictions. Notably, three features extracted from the DWI sequence held the highest importance, underscoring the critical value of functional diffusion information. Among these, DWI Imc1_glcm_logarithm was the most impactful predictor. The analysis further indicated that higher values of the top DWI and T2W texture features were consistently associated with an increased risk of SDM. This pattern suggests that greater intra-tumor heterogeneity, as quantified by these texture metrics, is a key imaging biomarker for metastatic potential.

## Discussion

Preoperatively identifying RC patients at an elevated risk of SDM is critical for individualized management strategies, optimizing the accuracy of metastatic detection, and facilitating timely lesion resection^31^. Furthermore, in cases where imaging modalities fail to detect occult metastases, for high-risk SDM patients undetectable by imaging, earlier identification allows for more proactive treatment measures and shorter follow-up intervals. Our multiparametric MRI-based radiomics nomogram, which integrates radiomics features with the clinical model, significantly enhances the predictive performance of SDM in RC patients, elevating the AUC from 0.83 to 0.94. The high specificity indicates that the model is reliable and can rule out false-positive and false-negative patients. We developed a clinical-radiomics nomogram as an individualized and visual tool to estimate the probability of SDM for newly diagnosed RC patients. DCA was used to determine its clinical benefit.

The main diagnostic tasks of radiomics should be the detection of synchronous and metachronous lesions^32^. Huang et al.^33^ revealed that the radiomics signature could help predict lymph node (LN)-positive patients with a C-index of 0.773 in the validation cohort, and the proposed clinical-radiomics nomogram was helpful in predicting LN involvement. Previous preliminary studies demonstrated that T2WI-based radiomics analysis can improve the prediction of SDM^27^. It is noteworthy that in our research, the T2W-only model showed a marked decrease in performance from the training set (AUC = 0.83) to the test set (AUC = 0.68). This underscores the importance of multiparametric integration and robust feature selection to enhance model reliability. Our model integrates DWI-derived features to quantify alterations in the tumor microenvironment. DWI enables microenvironment-level analysis of lesions that are invisible to visual inspection, providing quantitative insights beyond conventional imaging^34^. While Liu et al. reported an AUC of 0.827 for SDM prediction using T2WI alone, our clinical-radiomics model demonstrates superior discriminative ability, potentially explaining cases where T2WI features underperform in the detection of occult metastasis. Using the nomogram, clinicians can calculate the probability of SDM by incorporating selected radiomics features along with key clinical variables. For high-risk patients, strategies involving further imaging studies (including contrast-enhanced MR or FDG PET-CT) should be considered to detect additional metastatic lesions for personalized management.

Our results show that the nomogram provides predictive information about the synchronous distant metastasis (SDM) of rectal cancer (RC). Contrast-enhanced CT, MRI, and PET-CT are common imaging examinations for the preoperative diagnosis of synchronous liver metastases (SLM) in RC. However, the sensitivity and accuracy of these imaging techniques are not satisfactory^35–37^. According to one meta-analysis, the detection sensitivities of colorectal liver metastases in contrast-enhanced CT, routine MRI, and FDG PET-CT were 63%–80%, 76%–85.7%, and 51%–90%, respectively^38^. Many studies have shown that MRI has a higher accuracy compared to CT in diagnosing SLM of RC patients^39–41^, and recent consensus guidelines from the radiologic community recommend MRI for the preoperative evaluation of SLM^31,34^. Other studies have shown that some adverse features found on rectal MRI can identify patients with RC who are at a higher risk of distant metastasis^21,42,43^. Therefore, we developed a radiomics nomogram based on multiparametric MRI to predict the risk of SDM.

Screening for high-risk predictors would enhance the probability of early detection of SDM in RC patients. Typically, the clinicopathological predictors of SDM in CRC patients encompass the tumor site, depth of tumor invasion, lymph node status, vascular invasion, and tumor markers^44^. However, some of these predictors can only be obtained postoperatively, and thus, they are inappropriate for guiding preoperative treatment. Other studies have demonstrated that certain features of rectal MRI, such as extramural vascular invasion, T stage, and regional lymph node metastasis, are potential predictors^42,43^. However, these image features are subjective and qualitative, lacking quantitative assessment. In recent years, radiomics has been regarded as an advanced tool for evaluating tumor heterogeneity in tumor diagnosis and prognosis prediction. In this study, factors such as radiomics features, CEA, and CA19-9 levels were incorporated into multivariate logistic regression to construct a prediction model and a nomogram, and the research results are promising. Therefore, our analysis indicates that the radiomics nomogram combined with tumor markers was superior to the radiomics signature alone. It demonstrated high predictive performance for SDM in RC patients, and the AUC increased from 0.82 to 0.94. Moreover, the results were better than those reported in a previous study on a per-patient basis. In that study, the AUC values were 0.92 and 0.88 (for MRI readers), 0.80 and 0.82 (for CT readers), and 0.83 and 0.84 (for PET-CT readers)^45^. In this study, we constructed a primary RC-based radiomics nomogram using high-resolution T2WI and DWI of the rectum to predict SDM. On the training set, the AUC, accuracy, sensitivity, specificity, and 95% CI of the nomogram model were 0.93, 0.85 (114/134), 0.85 (55/65), 0.86 (59/69), and 0.89–0.96, respectively. The AUC, accuracy, sensitivity, specificity, and 95% CI of the nomogram model were 0.94, 0.89 (31/35), 0.88 (15/17), 0.89 (16/18), and 0.79–0.97, respectively. Therefore, based on clinical risk factors and radiomics characteristics, the proposed nomogram may serve as a valuable predictive tool for SDM in patients with RC. It can be readily employed to identify patients who require further whole-body imaging.

Radiomics features consist of multiple features, which are highly significant for selecting the optimal feature set through dimensionality reduction. In this study, we employed the LASSO method for feature screening and constructed a radiomics model based on an SVM. The application of LASSO for dimension reduction was aimed at enhancing the stability of the nomogram and facilitating a more robust overall analysis. The nomogram we constructed is more user-friendly for clinicians as it can extract information from T2WI and DWI and incorporates clinical risk factors. The radiomics nomogram is an auxiliary tool that can be used to identify and monitor patients with RC, when the group is divided into a low-risk group and a high-risk group, the high-risk group has a higher probability of developing SDM. Therefore, in a certain sense, the radiomics nomogram can serve as a clinically applicable and reliable detection tool for SDM in patients with RC. It is quick and easy to implement and helps to identify which patients will benefit from further imaging of distant metastases. Our nomogram offers practical benefits for clinical integration without incurring additional scanning or costs. It enables rapid risk stratification in the preoperative workflow, allowing for a more efficient allocation of subsequent diagnostic resources (e.g., PET-CT) to high-risk patients and enhancing cost-effectiveness. Thus, the model serves not only as a predictive tool but also as an aid for optimizing patient management pathways.

However, the current study still has some limitations. Firstly, the sample size in the single-institution retrospective analysis is relatively small. Moreover, selection bias, follow-up bias, and potential confounders like treatment heterogeneity and imaging protocol variations are inevitable. Secondly, this study lacks external validation. Therefore, an independent, large-scale multi-center trial is required to enhance the generalizability of the results. Thirdly, manual segmentation remains a source of variability and inefficiency. Future efforts should prioritize automated segmentation techniques and investigate causal modeling approaches—such as those exemplified by CausalSR^46^—to mitigate data bias and strengthen the generalizability of radiomics-based prediction.

## Conclusions

We developed a multiparametric MRI-based radiomics nomogram by combining tumor markers with imaging features to assist in predicting the presence of SDM in RC patients. The model demonstrated robust discrimination and calibration. This visualization tool can detect the probability of SDM and assist doctors in making clinical decisions.

## Supplementary Information

Below is the link to the electronic supplementary material.

Supplementary Material 1

Supplementary Material 2

## Data Availability

The data that support the findings of this study are not openly available due to sensitivity issues and can be obtained from the corresponding author upon reasonable request.

## Code availability

The code supporting the findings of this study is available under the following conditions.

Derived Research Code: The curated dataset containing extracted radiomics features, corresponding clinical variables, and patient outcomes generated during this study has been deposited in the Zenodo repository and is publicly available via DOI: 10.5281/zenodo.18098176.

Analysis Code: The custom Python code used for feature extraction, model training, and validation is publicly available in the GitHub repository at https://github.com/yimingfeitian/predicts-synchronous-distant-metastasis-in-rectal-cancer.
