# Supplementary material for: Multiparametric MRI radiomics nomogram predicts synchronous distant metastasis in rectal cancer
Source: Sci Rep. 2026 Jan 19;16:5759. doi: 10.1038/s41598-026-35973-w (PMC12894970; doi:10.1038/s41598-026-35973-w)

Multiparametric MRI radiomics nomogram predicts synchronous distant metastasis in rectal cancer

Hao Jiang[#]1, Wei Guo[#]2, Xue Lin1, Zhuo Yu3, Yudie Qin1, Zhongqi Sun1, Hongbo Hu1, Jinping Li1, Linhan Zhang4, Qiong Wu1, Huijie Jiang*1

1 Department of Radiology, The Second Affiliated Hospital of Harbin Medical University, Harbin, 150086, China

2 Department of PET/CT-MR, Harbin Medical University Cancer Hospital, Harbin, China

3 Hangzhou Lin ping research medical film technical service studio, China

4 Department of Nuclear Medicine, The First Affiliated Hospital of Harbin Medical University, Harbin, China

Appendix 1

Magnetic resonance imaging (MRI) examinations were conducted using a Discovery MR750w 3.0T MR scanner equipped with a phased-array coil, following a standardized protocol that captured oblique axial, sagittal, and coronal views. High-resolution oblique axial T2-weighted images and diffusion-weighted (DW) images were analyzed in the study. Before the MRI scan, an enema was administered using water to minimize artifacts caused by intestinal contents in the images. The oblique axial T2-weighted imaging (T2WI) was performed with the following protocol: repetition time (TR)/echo time (TE), 5,990 ms/125.7 ms; field of view (FOV), 20 cm; and 3.6-mm thickness with 0.3 mm slice spacing; the oblique axial diffusion-weighted imaging (DWI) was performed with the following protocol: TR/ TE, 4,881 ms/70.2 ms; FOV, 28 cm; 3.6-mm thickness with 0.3 mm slice spacing and b values of 0 and 800 s/mm^2.

Supplementary Table S1 Selected radiomics features and relevant coefficients

Signature	Features selected	Feature name	Coef
**DWI model**	8	Imc1_glcm_original	-0.092846
		Imc2_glcm_original	0.037184
		Imc2_glcm_logarithm	1.347927
		Imc2_glcm_squareroot	1.739902
		RobustMeanAbsoluteDeviation_firstorder_lbp-2D	0.008635
		Maximum_firstorder_lbp-3D-k	-0.038881
		Range_firstorder_lbp-3D-k	-0.065654
		10Percentile_firstorder_gradient	-0.010705
**T2W model**	8	ShortRunLowGrayLevelEmphasis_glrlm_original	0.086151
		ShortRunLowGrayLevelEmphasis_glrlm_logarithm	6.796077
		Maximum_firstorder_lbp-3D-k	-0.020915
		Range_firstorder_lbp-3D-k	-0.114862
		90Percentile_firstorder_wavelet-LHL	1.303794
		RobustMeanAbsoluteDeviation_firstorder_wavelet-LHL	-0.630481
		InterquartileRange_firstorder_wavelet-LHL	-0.589645
		10Percentile_firstorder_lbp-2D	-0.058816
**Radiomics model**	6	DWI_Imc1_glcm_original	-0.116921
		DWI_Imc1_glcm_logarithm	-1.864180
		DWI_Imc1_glcm_squareroot	-1.159935
		T2_ShortRunLowGrayLevelEmphasis_glrlm_original	0.064318
		T2_ShortRunLowGrayLevelEmphasis_glrlm_logarithm	4.639738
		DWI_RobustMeanAbsoluteDeviation_firstorder_lbp-2D	0.002514

glcm: Gray Level Co-occurrence Matrix; lbp: Local Binary Pattern; glrlm:Gray Level Run Length Matrix; Coef: coefficients.

Supplementary Note The Rad-score calculation formula of the three models is as follows:

Rad-score (DWI model) = Imc1_glcm_original*-0.092846
+ Imc2_glcm_original*0.037184
+ Imc2_glcm_logarithm*1.347927
+ Imc2_glcm_squareroot*1.739902
+ RobustMeanAbsoluteDeviation_firstorder_lbp-2D*0.008635
+ Maximum_firstorder_lbp-3D-k*-0.038881
+ Range_firstorder_lbp-3D-k*-0.065654
+ 10Percentile_firstorder_gradient*-0.010705

Rad-score (T2W model) =
ShortRunLowGrayLevelEmphasis_glrlm_original*0.086151
+ ShortRunLowGrayLevelEmphasis_glrlm_logarithm*6.796077
+ Maximum_firstorder_lbp-3D-k*-0.020915
+ Range_firstorder_lbp-3D-k*-0.114862
+ 90Percentile_firstorder_wavelet-LHL*1.303794
+ RobustMeanAbsoluteDeviation_firstorder_wavelet-LHL*-0.630481
+ InterquartileRange_firstorder_wavelet-LHL*-0.589645
+ 10Percentile_firstorder_lbp-2D*-0.058816

Rad-score (Radiomics model) = DWI_Imc1_glcm_original*-0.116921
+ DWI_Imc1_glcm_logarithm*-1.864180
+ DWI_Imc1_glcm_squareroot*-1.159935
+ DWI_RobustMeanAbsoluteDeviation_firstorder_lbp-2D*0.002514
+ T2_ShortRunLowGrayLevelEmphasis_glrlm_original*0.064318
+ T2_ShortRunLowGrayLevelEmphasis_glrlm_logarithm*4.639738

Supplementary Fig. S1. Confusion matrix for the nomogram model in the training set (a) and test set (b)

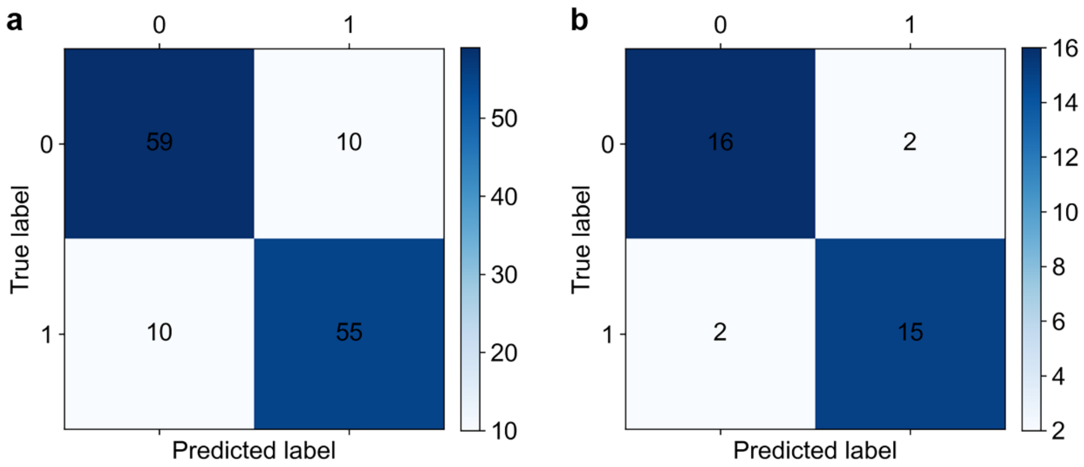

Supplement: Supplementary file 1 — Supplementary Material 1 [file 41598_2026_35973_MOESM1_ESM.pdf]
